# Similar but separate systems underlie perceptual bistability in vision and audition

**DOI:** 10.1038/s41598-018-25587-2

**Published:** 2018-05-08

**Authors:** Susan L. Denham, Dávid Farkas, Raymond van Ee, Mihaela Taranu, Zsuzsanna Kocsis, Marina Wimmer, David Carmel, István Winkler

**Affiliations:** 1 University of Plymouth, Cognition Institute and School of Psychology, Plymouth, PL4 8AA UK; 2 Institute of Cognitive Neuroscience and Psychology, Research Centre of Natural Sciences, Hungarian Academy of Sciences, H-1117 Budapest, Magyartudósok körútja 2 Hungary; 3 Institute of Psychology, Faculty of Humanities and Social Sciences, Pázmány Péter Catholic University, H-2087 Piliscsaba, Egyetem street 1 Hungary; 4 Radboud University, Donders Institute for Brain, Cognition and Behavior, Biophysics/85 PO Box 9010, 6500 GL Nijmegen, The Netherlands; 5 Leuven University, Department of Brain and Cognition, Tiensenstraat 102, 3000BE Leuven, Belgium; 6 Philips Research, Department of Brain, Behavior and Cognition, High tech campus, Bldg 34, 5656AE Eindhoven, The Netherlands; 7 University of Edinburgh, Department of Psychology, Edinburgh, EH8 9JZ UK

## Abstract

The dynamics of perceptual bistability, the phenomenon in which perception switches between different interpretations of an unchanging stimulus, are characterised by very similar properties across a wide range of qualitatively different paradigms. This suggests that perceptual switching may be triggered by some common source. However, it is also possible that perceptual switching may arise from a distributed system, whose components vary according to the specifics of the perceptual experiences involved. Here we used a visual and an auditory task to determine whether individuals show cross-modal commonalities in perceptual switching. We found that individual perceptual switching rates were significantly correlated across modalities. We then asked whether perceptual switching arises from some central (modality-) task-independent process or from a more distributed task-specific system. We found that a log-normal distribution best explained the distribution of perceptual phases in both modalities, suggestive of a combined set of independent processes causing perceptual switching. Modality- and/or task-dependent differences in these distributions, and lack of correlation with the modality-independent central factors tested (ego-resiliency, creativity, and executive function), also point towards perceptual switching arising from a distributed system of similar but independent processes.

## Introduction

Unravelling the perceptual strategies used by the brain to make sense of the world remains an ongoing challenge, not least because people differ both in their intrinsic makeup and life experiences which help to shape their individual information processing systems. A perceptual phenomenon which provides some help in this respect is that of perceptual bi- or multi-stability^1,2^. Qualitative changes in perceptual experience in response to an unchanging sensory input as well as consistent individual differences in perceptual behaviour^3–6^ can provide insights into perceptual grouping and decision-making processes. Although bistability exists in different modalities – visual, auditory, and even olfactory – it remains unknown whether perceptual switches in the different modalities are governed by a central mechanism or by separate, modality-specific systems. Similar fundamental properties of perceptual bistability are observed both in vision and audition^7,8^, suggesting that common processes may be involved. However, there is little evidence for cross-modal commonality at the individual level. Here, we show that perceptual switching in vision and audition arises from highly similar but independent sources.

The same principles of perceptual alternation, e.g., general properties of exclusivity, inevitability, and stochasticity^2^, and Levelt’s propositions^9,10^, are observed in many different visual paradigms^11–13^. Perceptual switching behaviour consistent with these principles is also observed in the auditory modality^14,15^. These principles have formed the basis for a number of models of perceptual bistability both in vision^16–18^ and audition^19^. However, in general the models are expressed at a rather abstract level, and are agnostic as to how their processes might map onto brain structures and processing systems.

Brain-imaging studies show a widespread network of areas (primarily in right hemisphere) that typically appear in transition-related contrasts in fMRI studies of visual bistability, including inferior frontal cortex (IFC), dorsolateral prefrontal cortex (DLPFC), frontal eye fields (FEF), temporoparietal junction (TPJ), and intraparietal sulcus (IPS)^20^; IPS was also identified in a fMRI study of auditory bistability^21^. However, there is controversy surrounding interpretation of these findings. Various fMRI studies (e.g.,^22–24^) identified a network of early visual and frontoparietal regions whose activity was time-locked to perceptual switches, but some more recent studies^25–29^ have suggested that at least the frontal activity may be related to response generation rather than perception. Studies manipulating parietal activity with transcranial magnetic stimulation (TMS) have demonstrated different causal roles for separate parietal sub-regions in perceptual switching, with stimulation of a posterior locus prolonging the time between switches^30^ and stimulation of a more anterior locus decreasing it^31,32^. As these parietal regions are not strictly visual, the results support the possibility of a distributed, high-level network involved in the control of bistability across modalities. However, application of TMS to non-visual bistability has not yet been reported.

Perceptual switching is typically characterised by the distribution of perceptual phase durations (i.e. periods during which one interpretation is experienced); generally reported as a gamma^9^ or log-normal distribution^33^. A recent meta-analysis by Cao, *et al*.^34^ showed, furthermore, that perceptual phase durations exhibit so-called scale free properties across many different visual and auditory paradigms. Therefore, they suggested that perceptual switching might best be explained by changes in the combination of discrete states across a finite set of independent processes, such as switches between up and down states in cortical columns; i.e., a highly distributed system in which the specifics depend on the brain areas involved in the task.

To date, three studies have investigated correlations between perceptual switching in visual and auditory tasks^7,14,35^. Small but significant within-individual correlations were reported between visual and auditory perceptual switching in one study^14^. In contrast, Pressnitzer and Hupé^7^ found that although the general properties of bistability were similar in the two modalities, the number of perceptual switches was not significantly correlated across modalities at the individual level. In their next study^35^, participants were required to report their perceptions of the visual and auditory bistable stimuli concurrently. They found no significant evidence that switching in one modality predicted switching in the other. To some extent these data favour interpretation in terms of a distributed system of perceptual switching, but the results so far are somewhat equivocal.

In the present study we aimed to compare perceptual switching behaviour within individuals using the visual ambiguous structure-from-motion^36^ and auditory streaming^37^ tasks. In the experiment reported in the main text, participants were required to classify their perceptions using two perceptual categories (bistability); in the supplementary material we report a three-category (multistable) version of the experiment.

Support for a distributed stimulus-driven system might be found if any of the commonalities are present by default and not as a result of some central process like attention. If a central system sensitive to top-down effects is an important source of commonality between visual and auditory perceptual switching, then we would expect to observe far higher correlations across modalities when participants are asked to bias their perception in some way, than in a neutral condition, because the instruction to bias leads to tighter top-down control, which would affect the central switching mechanism. A combination of top-down and stimulus-driven effects have been assumed in some previous models of bistability; e.g.^38,39^. Therefore, we correlated individual switching rates across modalities in three conditions, a Neutral condition, and two biased conditions (Hold – participants were asked to hold onto each percept for as long as possible, and Switch – participants were asked to switch as quickly as possible between alternative percepts).

Another possible central source of correlation would be some form of switching control centre generating switching signals that are fed back down to the sensory areas, as suggested by some of the brain imaging and manipulation studies, reviewed above. If there is such a modality-independent central switch generator involved (previous evidence has been specifically for vision), then we would expect the properties of perceptual switching in the two modalities to be very similar indeed, right down to the detailed level of perceptual phase distributions. Finding correlations between perceptual switching behaviour and modality-neutral central factors, such as ego-resiliency, creativity, or executive function, would also be consistent with a central, modality-independent source of switching.

Finally, a different source of commonality may lie at the microcircuit level, conceptually hypothesised previously in computational theories^16,17^, and recently supported by the analysis and model of Cao, *et al*.^34^. Common microcircuit properties across the different sensory modalities within an individual could produce similar switching properties across tasks and modalities. However, while there may be strong similarities in perceptual switching behaviour overall, we would expect there to be observable differences at the detailed level of perceptual phase distributions. To distinguish between the central and distributed system hypotheses, we analysed the phase duration data in detail. First, we established the type of distribution that best characterised the phase durations in each modality. Next, we calculated the parameters describing those distributions. Finally, at the most detailed level of comparison, we examined the relationships between successive phase durations at a range of lags.

## Results

### Condition and modality

**Figure 1:**
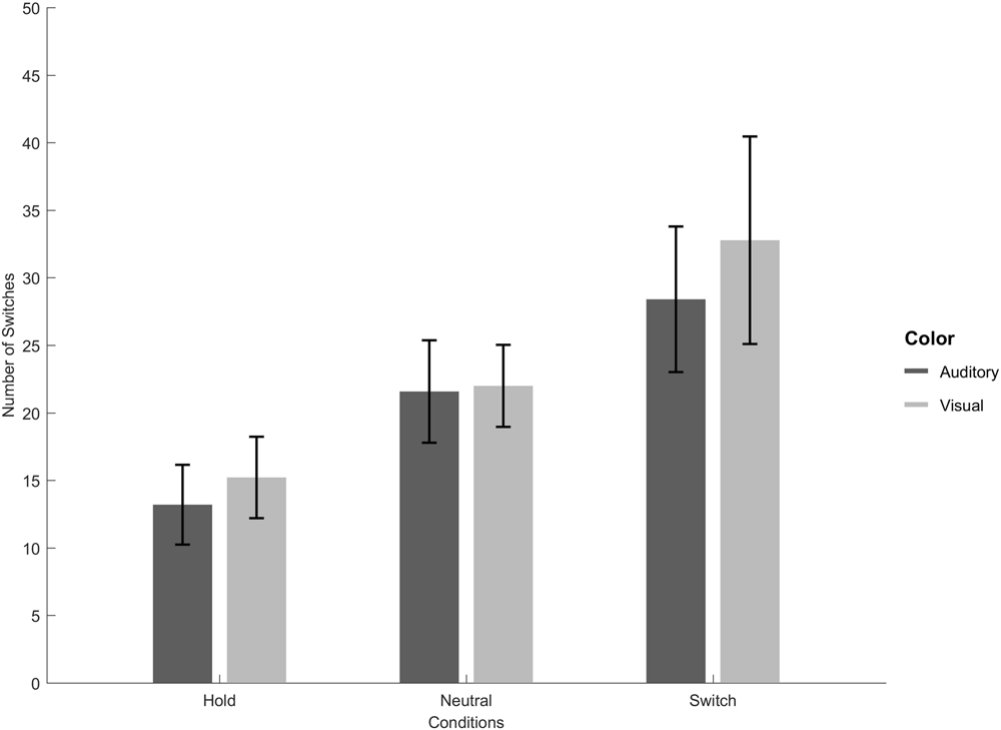
Mean number of perceptual switches during a 180-second block in each condition and modality. Error bars indicate 95% confidence intervals.

The influence of the attentional manipulation and modality on the average number of switches was assessed using a 2 (modality: visual, auditory) × 3 (condition: Neutral, Hold, Switch) repeated-measures analysis of variance (rmANOVA; the Greenhouse-Geisser correction was applied to violations of sphericity). The main effect of condition was significant (*F*(2,64) = 27.182, *p* < 0.001, *η*^2^_partial_ = 0.459, ε = 0.581). This shows that participants were able to bias their perception according to instructions, leading to longer durations in the Hold condition and shorter durations in the Switch condition, compared to the Neutral condition (Fig. 1). Neither the effect of modality (F(1,32) = 2.060, p = 0.161) nor the modality/condition interaction (F(2,63) = 1.252, p = 0.287) were significant. Thus, participants switched similarly across modalities (possibly due to the choice of stimulus parameters rather than inherent cross-modal commonalities; e.g., in visual bistability (Necker cube), switching rates decrease with increasing stimulus size^38^, in auditory steaming, switching rates decrease with increasing frequency difference and with decreasing presentation rate^40^), and the effect of attentional bias was similar for the two modalities.

### Correlations across modalities

**Figure 2:**
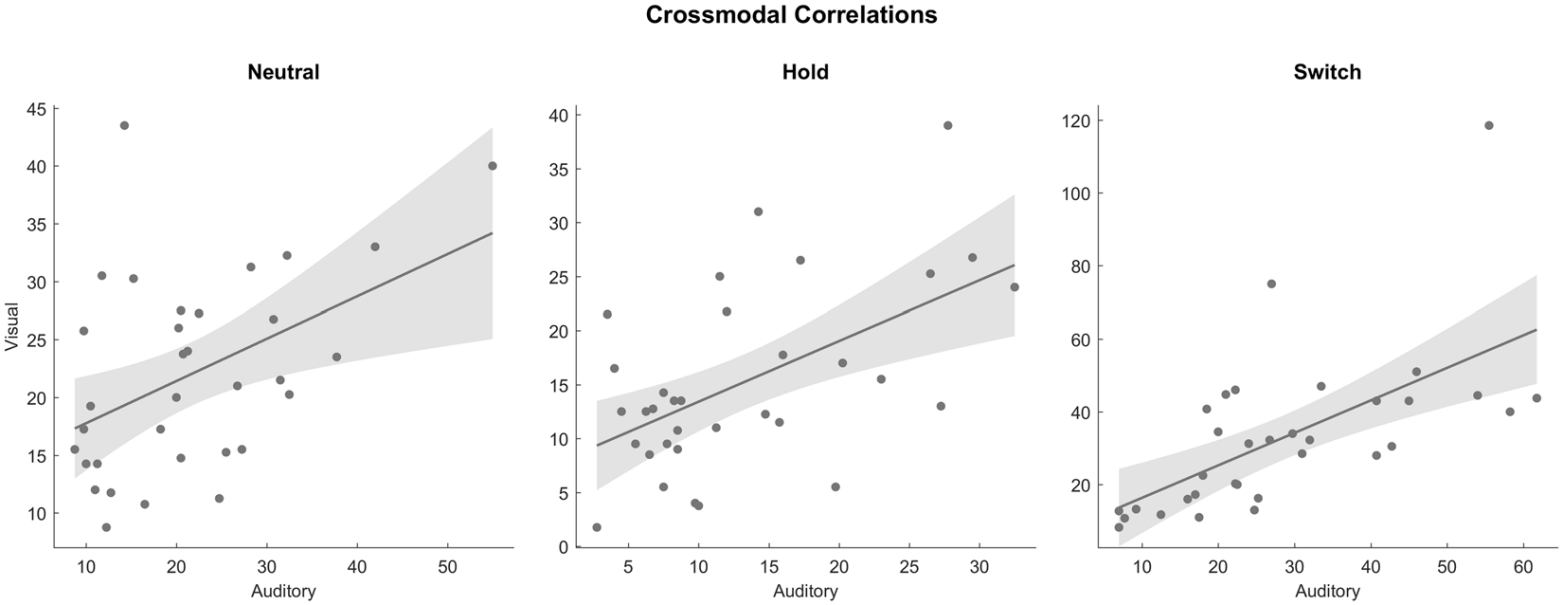
Correlations in the number of perceptual switches across modalities, separately for each condition; shading indicates 95% confidence intervals of the slope of the regression line.

Cross-modal relationships between number of switches were tested with Pearson correlations separately for each condition (Fig. 2). Correlations between the two modalities were significant in all three conditions: Neutral (*r*(33) = 0.456, *p* = 0.008, CI_95_ = 0.134–0.691), Hold (*r*(33) = 0.550, *p* = 0.001, CI_95_ = 0.255–0.752), and Switch (*r*(33) = 0.626, *p* < 0.001, CI_95_ = 0.361–0.798). The correlation coefficients observed for the Hold (*z* = −0.490, *p* = 0.624) and Switch (*z* = −0.940, *p* = 0.347) conditions did not significantly differ from those of the Neutral condition. The correlation observed in the Neutral condition is not significantly different (*z* = 0.238, *p* = 0.782) from the auditory-visual correlation (*r*(23) = 0.400, *p* = 0.060, *r*^2^ = 0.160) reported by Pressnitzer and Hupé^7^. No significant correlations were found between the number of switches in the Neutral condition and any measures in the creativity, ego-resiliency, and Stroop tasks.

### Individual consistency

We restricted the rest of our analyses to the Neutral data as the correlations between switching in the visual and auditory task did not differ between the neutral and biased conditions and there was no modality × bias interaction in the ANOVA, allowing us to assume that similar processes are at work in the neutral and biased conditions.

To explore individual consistency in switching across modalities, percepts were first reorganized into dominant/non-dominant categories (i.e., percepts were relabelled block-by-block according to their dominance to allow comparisons between the two modalities). We then constructed participant transition matrices^41^ from the relabelled data (see Supplementary Material for details). Intra-modal consistency was measured as the Kullback-Leibler (K-L)^42^ divergence between participants’ auditory and visual transition matrices. Inter-participant consistency was measured by comparing the K-L distances between a participant’s transition matrices and the transition matrices of all other participants. The distributions of intra-modal and inter-participant distance measures were compared using a one-tailed Wilcoxon’s Rank Sum test. The result of the test (*z* = −2.913, *p* = 0.002) indicates that participants’ perceptual switching behaviour is more similar across the two modalities (*M* = 0.042, CI_95_ = 0.028–0.057) than the variation across participants (*M* = 0.093, CI_95_ = 0.088–0.098). In short, participants responded consistently within and across modalities.

### Comparison of the distributions of phase durations across modalities

**Figure 3:**
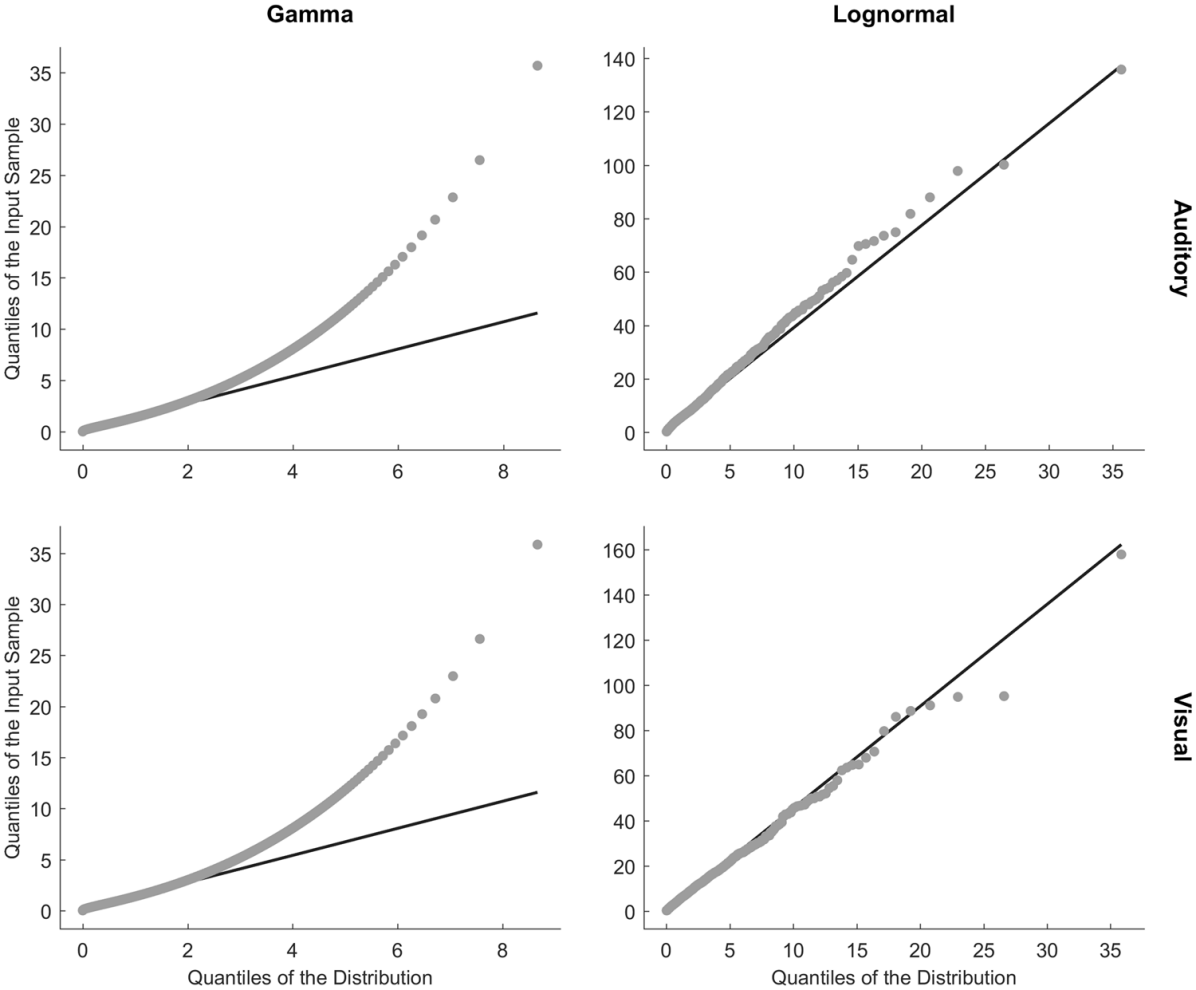
QQ-plots of gamma (left) and log-normal (right) distributions for phase durations from the Neutral conditions in the auditory (upper row) and visual (lower row) modalities.

Raw phase durations from the Neutral condition were pooled across participants separately for each modality. First, we tested whether the distribution of the phase durations was gamma or log-normal. Examination of Q-Q-plots^43^ indicated that the log-normal distribution fits our data better than the gamma distribution (Fig. 3). The Akaike Information AIC^44^, also indicated that the log-normal distribution fits the data better than gamma for both the auditory (AIC_log-normal_ = 16474, AIC_gamma_ = 17024) and visual (AIC_log-normal_ = 17070, AIC_gamma_ = 17504) modalities.

**Figure 4:**
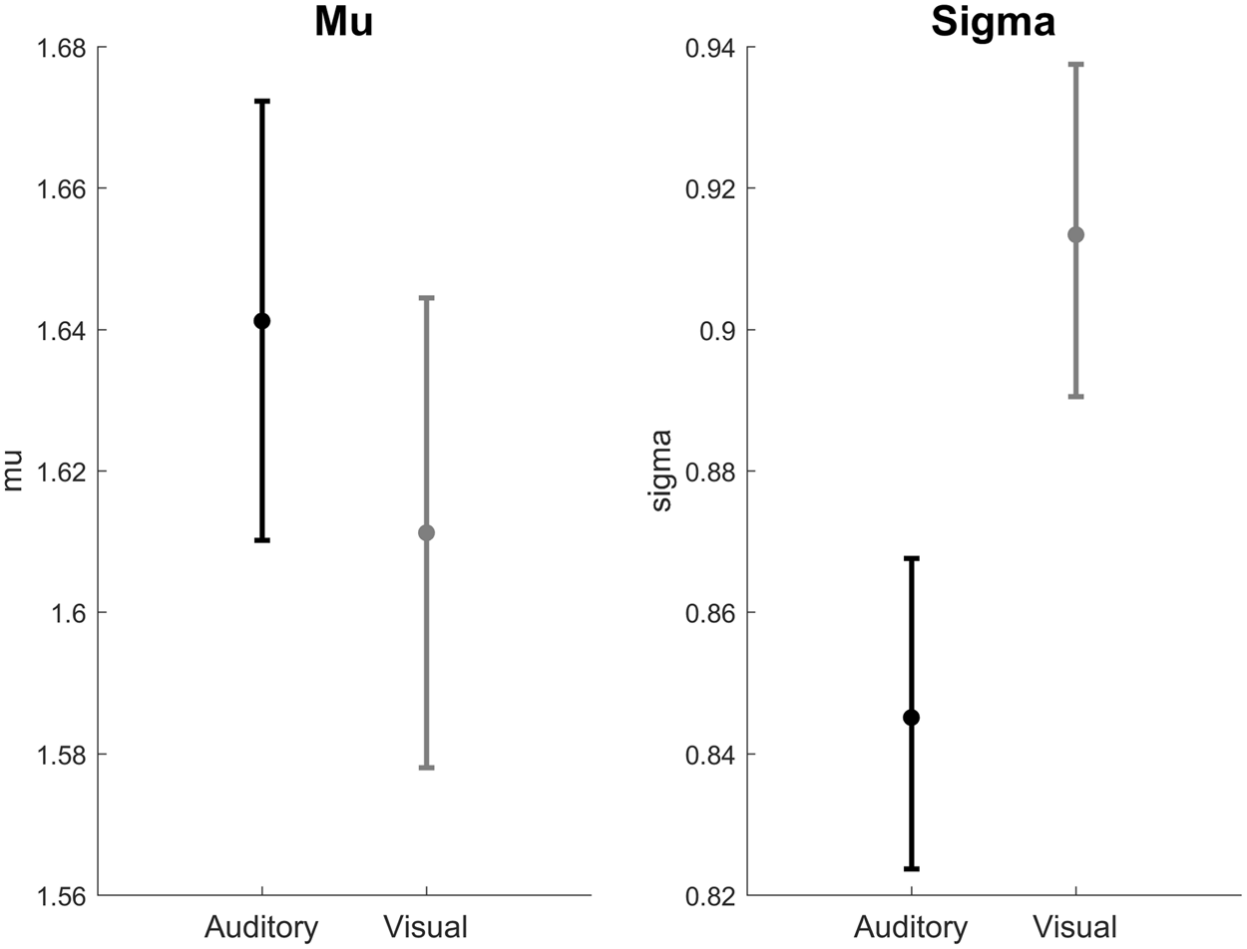
Mu and Sigma parameters of the log-normal distribution with 95% confidence intervals.

We then compared the auditory and visual phase distributions in two steps. First, phase distributions from the two modalities were compared using a Two-Sample Kolmogorov-Smirnov test. The result indicated that the auditory and visual distributions were significantly different (*D* = 0.047, *p* = 0.003). Second, to examine the factors underlying this difference, the mu and sigma parameters of the log-normal distributions (which determine the central tendency and variance, respectively), were calculated for phase durations separately for the auditory and visual modalities with 95% confidence intervals (Fig. 4). While the confidence intervals of the mu parameter overlap across the two modalities, the confidence intervals of the sigma parameter do not. This shows that although phase durations in the auditory and visual modalities can both be described by the same type of distribution (log-normal), the details of the distributions are different.

### Correlations between successive phases

Relationships between the duration of each perceptual phase and the durations of perceptual phases at one, two and three lags were tested with a mixed-level linear regression where participant identity was included as a random effect. Raw phase durations from the Neutral condition were log_10_-corrected in accordance with their log-normal distribution. This allowed us to meet not only the normality but also the linearity and heteroscedasticity assumptions of the test. All lags were significant in the auditory modality, whereas non-significant models were observed for the visual modality in half of the cases. The explained variance (R^2^) is higher, whereas the Akaike (AIC) and Bayesian Information Criterion (BIC) values are lower for the auditory modality compared to the visual modality; see (Table 1).

**Table 1:** Relationship between successive phase durations in the auditory and visual modalities.

Modality	Transition	Lag	R^2^	AIC	BIC	Unstandardized *b*	*t*	*r*
Auditory	D/ND	#1	20.27%	512.75	533.10	0.249 (0.197–0.300)	9.516***	0.450
	*N* = 1198	#2	20.34%	625.13	645.48	0.186 (0.132–0.240)	6.775***	0.451
		#3	13.71%	583.16	603.52	0.092 (0.039–0.144)	3.407**	0.370
	ND/D	#1	21.36%	712.49	732.89	0.240 (0.183–0.298)	8.208***	0.462
	*N* = 1213	#2	13.97%	578.63	599.03	0.155 (0.100–0.209)	5.588***	0.374
		#3	20.30%	653.81	674.21	0.153 (0.096–0.209)	5.337***	0.451
Visual	D/ND	#1	14.85%	829.93	850.37	0.149 (0.100–0.199)	5.940***	0.385
	*N* = 1222	#2	5.77%	1158.48	1178.91	0.239 (0.184–0.293)	8.652***	0.240
		#3	13.48%	909.05	929.48	−0.023 (−0.074–0.028)	−0.878	0.361
	ND/D	#1	10.68%	1147.29	1167.75	0.147 (0.085–0.208)	4.648***	0.327
	*N* = 1231	#2	14.05%	885.74	906.21	0.060 (0.005–0.116)	2.131*	0.375
		#3	8.29%	1151.43	1171.89	0.037 (−0.025–0.098)	1.156	0.288

“Transition” refers either to the Dominant/Non-Dominant (D/ND) or the Non-Dominant/Dominant (ND/D) transitions. “Lag” refers to the number of intervening percept durations: for example, lag 1 refers to the correlation with the percept duration immediately following each individual duration. R^2^ refers to the explained variance of the model, whereas AIC and BIC refer to the Akaike or Bayesian Information Criterion, respectively. “Unstandardized b” refers to the slope of the model with CI_95_ values included in parenthesis. “t” refers to the t-test examining the slope’s difference from zero and asterisks are indicating the level of significance (***p < 0.001, **p < 0.01, *p < 0.05). “r” refers to the correlation coefficient between the two phases estimated from the R^2^.

## Discussion

Our aim was to investigate commonalities between visual and auditory bistability using two well-studied exemplar tasks, visual ambiguous structure-from-motion and auditory streaming. We found strong similarities in the perceptual switching behaviour of individuals across modalities when viewed at a coarse level of description, but differences when viewed at a finer level of detail.

The main evidence for similarity comes from the strong correlations between the number of switches participants reported over the same duration in the visual and auditory tasks in all three conditions (Neutral, Hold and Switch). The correlations we found in the Neutral condition do not differ significantly from those reported by Pressnitzer and Hupé^7^, although in our study the correlation reached statistical significance, presumably thanks to the greater statistical power afforded by our larger sample. In accord with this finding, comparisons of the transition matrices (which can be used to characterise perceptual switching behaviour at a finer level of detail than the gross number of perceptual switches Denham, *et al*.^41^) showed that individual participants were consistent in their behaviour across blocks and across modalities, despite the well-known stochasticity of perceptual bistability. Furthermore, attentional manipulations affected perceptual switching similarly in both modalities. While the visual-auditory correlations were numerically larger when attentional manipulations were introduced, the differences between the correlations found in the different conditions were not significant, showing that the correlations observed are unlikely to arise principally from some attention-related top-down effect.

The distribution of phase durations in the two modalities was also quite similar. However, in contrast to the assumption of the gamma distribution used in many previous studies (e.g.,^2,9,18,45,46^) we observed a log-normal distribution in both modalities. Our observations are consistent with the findings of Rubin and Hupé^47^ and Lehky^33^ who also noted that their data was often better fit by a log-normal distribution. While a single, stochastic process would yield a gamma distribution, a log-normal distribution suggests that within each modality, switching is triggered by a multiplicative combination of a set of independent stochastic processes. This provides quite tight constraints on possible architectures. Recent theoretical considerations by Cao, *et al*.^34^ have shown that the distributions observed in many bistability experiments are better explained by the combination of a finite set of independently switching processes. The generalised Ehrenfest process they used in their analysis yields a log-normal-like distribution. Our findings are thus consistent with their proposals and have important implications for understanding and modelling the mechanisms underlying perceptual bistability.

Differences between the two modalities were found when the log-normal distributions characterising the phase durations in each modality were examined in more detail. Firstly, we found that the distributions were different (two-sample Kolmogorov Smirnov test). Secondly, we found that while the means of the distributions were similar, the spread (sigma) was not. Together, these findings support the hypothesis that perceptual switching in the two tasks depends on similar but distinct processes.

A similar conclusion emerges from considering the relationships between successive perceptual phases. Successive phase durations have often been assumed to be independent (i.e. consistent with phase durations having a gamma distribution)^9^, and this has influenced both theories and models of bistability (e.g.^45,48^). However, more recent work has shown that there are small but significant correlations between successive phases both in vision^45^ and audition^49^. The relationships we found were larger than those reported previously, likely because of differences in the analysis methods (see the Supplementary material for more details). The finding of significant correlations between successive phase durations points towards some form of memory with longer duration than considered in previous modelling work, something that may be interesting to explore in future work. The relationships between successive phases were higher for the auditory than for the visual data. This reinforces the emerging conclusion that while the very strong similarities between perceptual bistability in vision and audition imply that perceptual switching is generated in a very similar way in the two modalities, nevertheless, the precise source of switching differs.

There was no correlation between perceptual switching and any of the modality-neutral central factors tested (creativity, ego-resiliency, and executive function). While the absence of correlations with these tasks is not evidence of a distributed system, these findings certainly provide no support for a unified central source of switching. Since we have only used one exemplar task in each modality, we cannot distinguish between the existence of modality-dependent sources of switching, or a more distributed system in which switching arises from task-dependent sources. However, the log-normal distribution of perceptual phases that we found does point toward the latter interpretation. We also make no specific claims about what the underlying mechanisms might be; adaptation, increased inhibition, increased noise are all possibilities, but our experiment was not designed to distinguish between them. Thus, one possible extension of the current research is to test what roles each of these mechanisms might play in a distributed system such as that indicated by our results.

## Conclusion

Fundamentally similar properties characterise both auditory and visual perceptual bistability suggesting that the way perceptual switching is generated is very similar in both modalities. However, differences in the details of the phase distributions argue against a modality-independent central switch generator. Rather, they suggest a more distributed system in which switching is generated in very similar ways in different brain areas. Individual consistency within and across modalities, as well as the fact that the best fit function for describing the distribution is log-normal, suggest that generic circuit properties rather than purely stochastic processes, may lie at the heart of the switching process.

## Method

### Experimental Design

We adopted a mixed design in which each participant took part in one session. A complete session consisted of preliminary procedures followed by a period of training and three experimental conditions interleaved with a set of supplementary tasks; the experimental design is summarised in Table 2 below. Experimental conditions were distinguished by the task instructions given to participants: Neutral (should not try to influence their perceptions), Hold (should try to hold onto each percept they experienced for as long as possible), and Switch (should try to switch to a new percept as quickly as possible). Each condition comprised eight stimulus blocks, four visual (V) and four auditory (A). In the visual task, participants were asked to report the direction of motion of the front face of a rotating sphere (ambiguous structure-from-motion), while in the auditory task, they were asked to report on the perceptual grouping of tones in a sequence (auditory streaming). In the experiment reported here, participants were asked to report their perceptions using two perceptual categories. In the supplementary material we report a similar experiment in which participants were asked to report their perceptions using three perceptual categories; the principal effects are the same for both.

**Table 2:** Experimental design, showing the eight stages in an experimental session.

Stage	Activity	Description
1	Preliminary steps	Consent, handedness questionnaire^54^, hearing check
2	Training	Response categories
	-Visual task	LEFT, RIGHT
	-Auditory task	INTEGRATED, SEGREGATED
3	Test Condition 1: Neutral	8 stimulus blocks: VVVVAAAA (AAAAVVVV)
4	Supplementary activity	Ego-resiliency questionnaire, (creativity questionnaire, Stroop task)
5	Test Condition 2: Hold (Switch)	8 stimulus blocks: VVVVAAAA (AAAAVVVV)
6	Supplementary activity	Creativity questionnaire (Stroop task, ego-resiliency questionnaire)
7	Test Condition 3: Switch (Hold)	8 stimulus blocks: VVVVAAAA (AAAAVVVV)
8	Supplementary activity	Stroop task (ego-resiliency questionnaire, creativity questionnaire)

The order of the following was counterbalanced across participants: a) modality ordering of stimulus blocks, VVVVAAAA or AAAAVVVV, b) biased test conditions Hold/Switch in stages 5 and 7, c) supplementary task order ego-resiliency/creativity/Stroop in stages 4, 6 and 8.

### Participants

The study was run at two separate locations (Hungary: Research Centre for Natural Sciences of the Hungarian Academy of Sciences (RCNS); U.K.: University of Plymouth (UoP)), partly for practical reasons, and partly to reduce the risk of biasing the data by the specifics of the labs or their personnel. A total of 44 adults participated in this study (RCNS, 24 adults: 18 females, M_age_ = 21.5, SD_age_ = 1.98; UoP, 20 adults: 16 females, M_age_ = 22.9, SD_age_ = 9.52). The study was approved by the local ethics committee (Hungary: Unified Committee for Psychological Research Ethics (EPKEB); U.K.: Faculty of Health and Human Sciences Research Ethics Sub Committee, University of Plymouth). The research was performed in accordance with relevant regulations. Informed consent was obtained from each participant prior to beginning the experiment. All participants had normal or corrected-to-normal vision and normal hearing, and gave written informed consent. Participants received modest payment or course credits in return for their participation.

### Training

#### Visual Task

Participants viewed 500 dots (each subtending a viewing angle of 4.7 arcmin) projected onto a computer screen. The dots’ position changed from one frame to the next as if they were located at random positions on a rotating sphere, which subtended a viewing angle of 3.3 degrees. A chin rest was used to fix the distance of the head relative to the screen. At RCNS, the visual stimuli were presented on a Samsung 17″ TFT 740B screen with a resolution of 1280 × 1024 pixels, and at UoP on a Dell screen with a resolution of 1920 × 1080 pixels; stimulus parameters were adjusted to generate the same size sphere (same viewing angle) for the same screen to chin-rest distance. The virtual sphere rotated about a central vertical axis at an angular velocity of 75 degrees/second. Due to structure-from-motion effects, the moving dots create a vivid impression of a three-dimensional rotating sphere^36^. Because there are no depth cues indicating which dots belong to the front or the back of the sphere, the direction in which the sphere rotates is ambiguous, and alternates periodically.

**Figure 5:**
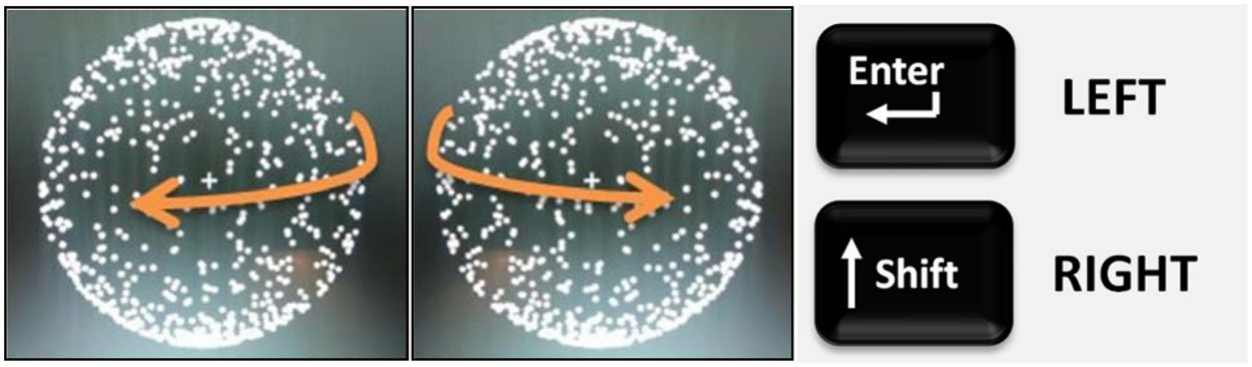
Mnemonics for the perceptual interpretations of the ambiguous structure-from-motion stimulus; LEFT, RIGHT and the key assignment.

Participants were instructed to report LEFT (by holding down a key) for as long as they perceived the front face of the rotating sphere moving leftwards, and RIGHT (by holding down a different key) for as long as they perceived it moving rightwards. These interpretations were demonstrated to participants using disambiguated examples (see Training Procedure, below). The corresponding mnemonics shown in Fig. 5 were used as reminders. The “Enter” and “Shift” keys located on the right-hand side of a standard computer keyboard were used as response keys, with key-response assignment counterbalanced across participants.

#### Auditory Task

Participants listened to a cyclically repeating sequence of tones, ordered low-high-low, with a brief gap before repeating (LHL_LHL_..), according to the auditory streaming paradigm^37^. Sinusoidal tones of 75 milliseconds (ms) duration were used; the frequency of the L tone was 400 Hz and the H tone was 504 Hz, a difference of 4 semitones. The stimulus onset asynchrony (SOA, onset to onset time interval) was 125 ms. The sounds were delivered through Sennheiser HD600 headphones at both locations. Most commonly, listeners either perceive this sequence as if all tones belong together, termed INTEGRATED (i.e., they form a single coherent sound stream with a typical galloping rhythm caused by the triplet pattern, or they hear the tones splitting apart into two separate isochronous streams of sounds, L_L_L.. and H___H___H.., termed SEGREGATED.

**Figure 6:**
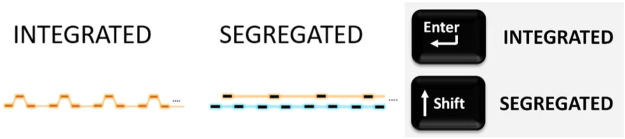
Mnemonics for the perceptual interpretations of the tone sequence; INTEGRATED, SEGREGATED, and the key assignment.

Participants were instructed to report INTEGRATED (by holding down a key) for as long as they perceived a single coherent sound stream and SEGREGATED (by holding down a different key) for as long as they perceived two streams, one containing low and the other high tones. These interpretations were demonstrated to participants using disambiguated examples (see Training Procedure, below). The corresponding mnemonics shown in Fig. 6 were used as reminders. The “Enter” and “Shift” keys located on the right-hand side of a standard computer keyboard were used as response keys, with key-response assignment counterbalanced across participants.

#### Training Procedure

Training in the two modalities was carried out separately, using a standardized procedure to ensure consistency. First, the perceptual categories were explained and demonstrated using stimuli in which the relevant category was disambiguated (visual disambiguation: to bias perception towards LEFT (RIGHT), luminance of rightward (leftward) moving dots was reduced; auditory disambiguation: to bias perception towards INTEGRATION (SEGREGATION), frequency difference between L and H tones was reduced (increased) to 1 (H = 424 Hz) and 10 (H = 713 Hz) semitones, respectively). Next, participants practiced continuously responding to 60-second versions of the test stimuli to each of which a short segment of a disambiguated sequence was concatenated; feedback on the proportion of time they correctly categorised the disambiguated section was provided. They also practised categorising sequences formed only from concatenated disambiguated stimuli, with feedback on the proportion of correct categorisation for each segment. Once participants understood and could easily categorise their perceptions, they proceeded to the test phase. No participant was rejected because they failed to understand the training requirements.

### Testing

The main experiment consisted of three conditions. In each condition, there were four contiguous 180-second visual blocks and four contiguous 180-second auditory blocks (VVVVAAAA or AAAAVVVV), with order counterbalanced across participants. At the end of each test block, there was an eight-second long disambiguated segment randomly chosen from LEFT, RIGHT (for visual) or INTEGRATED, SEGREGATED (for auditory). Responses to the disambiguated segments were used to monitor participant performance. A key press initiated the start of each block, so it was possible for participants to take short breaks between blocks. The initiating key press triggered an instruction screen sequence that prepared them for the block; this consisted of a 1 s blank screen, followed by a 10 s instruction screen showing category mnemonics and key assignment, then a 2 s blank screen followed by a 2 s central fixation cross before the stimuli for the block commenced.

#### Conditions

Conditions were defined by differences in the task instructions. In the Neutral instruction condition (which always ran first, to avoid carry-over effects from the other two conditions), participants were asked to report their perceptions without trying to influence them in any way. In the Hold condition, participants were asked to report their perceptions while at the same time trying to hold onto each percept for as long as possible. In the Switch condition, participants were asked to report their perceptions while at the same time trying to switch to a new percept as quickly as possible. The order of the Hold and Switch conditions were counterbalanced across participants. Instruction screens reminded participants of their current task.

### Data extraction

Perceptual reports were recorded by polling the key status every 10 ms for the duration of the stimulus, and the resulting data were processed to extract the sequences of continuous periods during which the same perceptual category was reported. This resulted in a sequence of perceptual phases together with the start time for each, relative to the start of the stimulus. The reports from the disambiguated segments were extracted separately and used as a measure of how well a participant understood the perceptual categories and the key assignments. To allow for a delay in reacting to the stimulus change^50^, the first two seconds of each disambiguated segment were ignored. Any participant who scored an average of less than 30% in a category, or less than 60% over both categories was excluded from further analysis. 11 participants were excluded from the analysis based on poor categorisation of disambiguated segments. Exclusion is based on a previous study^51^ showing, for the auditory task, that participants who correctly labelled the disambiguated segments showed greater internal consistency overall. The analysis reported here is based on 33 participants (25 females; 19–25 years; *M*_age_ = 21.45, *SD*_age_ = 1.82). The rejection rate is similar to previous studies using the same procedure^52,53^. For the analysis, we used the number of perceptual switches in each block and the phase durations.

### Supplementary tasks and data analysis

Full details of each of the supplementary tasks (the ego-resiliency and creativity questionnaires, and the Stroop task) and the data analysis are reported in the Supplementary Material.

## Electronic supplementary material

Supplementary Information
