# Supplementary material for: Similar but separate systems underlie perceptual bistability in vision and audition
Source: Sci Rep. 2018 May 8;8:7106. doi: 10.1038/s41598-018-25587-2 (PMC5940790; doi:10.1038/s41598-018-25587-2)
Supplement: Supplementary file 1 — Supplementary Information [file 41598_2018_25587_MOESM1_ESM.pdf]

**Supplementary Material for:**

# Similar but separate systems underlie perceptual bistability in vision and audition

Susan L. Denham[1], Dávid Farkas[2,3], Raymond van Ee[4,5,6], Mihaela Taranu[1], Zsuzsanna Kocsis[2], Marina Wimmer[1], David Carmel[7], István Winkler[2]

[1] University of Plymouth, Cognition Institute and School of Psychology, Plymouth PL4 8AA, UK

[2] Institute of Cognitive Neuroscience and Psychology, Research Centre of Natural Sciences, Hungarian Academy of Sciences, H-1117 Budapest, Magyartudósok körútja 2, Hungary

[3] Institute of Psychology, Faculty of Humanities and Social Sciences, Pázmány Péter Catholic University, H-2087, Piliscsaba, Egyetem street 1, Hungary

[4] Radboud University, Donders Institute for Brain, Cognition and Behavior, Biophysics/85 PO Box 9010, 6500 GL, Nijmegen, The Netherlands

[5] Leuven University, Department of Brain and Cognition, Tiensenstraat 102, 3000BE, Leuven, Belgium

[6] Philips Research, Department of Brain, Behavior and Cognition, High tech campus, Bldg 34, 5656AE, Eindhoven, The Netherlands

[7] University of Edinburgh, Department of Psychology, Edinburgh EH8 9JZ, UK

This document contains further details of the methods for the experiment reported in the main paper and an outlier analysis and discussion of the successive phase duration correlations for that experiment. It also describes a second experiment, much like that reported in the main paper, in which the number of perceptual alternatives available to participants was increased to three.

## Method

This section contains details of the supplementary Stroop task, the ego-resiliency and creativity questionnaires, and the data analysis.

### Supplementary tasks

#### *Stroop task*

Participants' inhibitory control was measured using a computerized version of the Stroop task [1]. Words, coloured red, green or blue, were presented on the same screen used for the experiment, and subtended a vertical angle of 0.9 degrees and a horizontal angle of 3.3-4.7 degrees (the horizontal angle changing with word length). Participants were instructed to respond as quickly and accurately as possible using the arrow keys with their dominant hand on a standard computer keyboard mapped as follows: ↑ for red, ← for blue, and → for green. Stimuli were shown on the screen until one of the response keys was depressed. Each response was followed by a blank screen for 250 ms.

The task consisted of three conditions. In each condition, there were 60 trials. Condition 1 was the neutral-word condition; the names of the three colours (red, blue and green) appeared on a white screen written in black. Participants were required to press the arrow

key corresponding to the colour name. Condition 2 was the neutral-colour condition; four X's appeared on the screen in one of the three colours and participants had to press the arrow key corresponding to the colour of the X's. Condition 3 was the congruent-incongruent condition; colour names appeared on the screen either in the corresponding colour (e.g. "red" coloured red) – Congruent trial, or in one of the other two colours (e.g. "red" coloured blue) – Incongruent trial. Participants were required to press the arrow key corresponding to the colour of the letters (not the word). There were equal numbers of Congruent and Incongruent trials, with order separately randomised for each participant. The Congruent/Incongruent condition received two blocks of 60 trials.

The Stroop interference effect was measured as the difference between the median reaction times of the correct responses in the congruent condition and the median reaction time of the correct responses in the incongruent conditions. Thus, a smaller reaction time difference indicates stronger inhibitory control of a prepotent response.

***Ego-resiliency questionnaire***

The paper-based ER89 questionnaire [2] was used to measure ego-resiliency (ER) at UoP. ER89 has 14 items (e.g. ("I like to do new and different things" and "My daily life is full of things that keep me interested"). Participants were instructed to indicate to what extent each item applied to them on a four-level Likert scale from "Does not apply to me at all" to "Applies to me very much". In the Hungarian version[3], ER was measured using 11 items from the ER89 questionnaire. For consistency the UoP scores were calculated using the same 11 items. Cronbach's α was 0.671 across all participants. The ER score for each participant is calculated by averaging the score of the eleven items per participant.

### *Creative behaviour questionnaire*

The paper-based Biographical Inventory of Creative Behaviours (BICB) [4] was used as a measure of individual creativity. BICB is a 34-item questionnaire, in which participants are instructed to indicate whether they have participated in various creative activities (e.g., “Invented a game or other form of entertainment”, “Composed a poem”, “Started a club, association or group”). Participants were asked to tick all the items that applied to them. The BICB score for each participant is the number of items they ticked.

## Data Analysis

To ensure compatibility with previous research, durations of phases during which no response was recorded were merged with the following perceptual pattern. (22 (20)[1] participants did not report any no-responses longer than 300ms, and for those that did, there was no difference in the probability of no-response across conditions and modalities). The number of switches was calculated separately for each block and the scores were averaged across participants separately for each condition and modality. This measure was used to test the effect of attentional manipulations with a repeated measures ANOVA and the connection between modalities with Pearson’s correlation. Correlations were compared to each other using Fisher’s r to z test[5]. For all further analysis, only the data from the Neutral condition were used. The reason for this is that our main effect (the correlation between auditory and visual modalities) was not different between voluntary and neutral

[1] X(Y) indicates the number of participants in the experiment reported in the main paper (and in the experiment reported in this document).

conditions. Thus, the same effects should be observed in further analyses. Repeating all analyses for each condition complicates the results unnecessarily.

For the phase distribution analysis, the last percept in each block was omitted from the analysis, because they were terminated by the end of the block and not by the participant. Phase durations were pooled from all participants separately for each modality. Quantiles of the data were tested against quantiles of gamma and lognormal distributions with Q-Q plots[6] separately for the two modalities. Phase distributions from the auditory and visual modality were compared using a Two Sample Kolmogorov-Smirnov test [7]. In addition, the parameters of the distributions were estimated from the data with 95% confidence intervals and the two modalities were also compared based on the similarity of their parameters.

For the phase correlation and intra-modal consistency analysis, percepts were relabelled block-by-block according to their dominance to allow more detailed comparisons between the two modalities; the percept with the larger proportion in a block was labelled as dominant and the other was labelled as non-dominant. This was required to be able to compare the perceptual patterns between modalities. For the phase correlation analysis, we analysed transitions starting from dominant to non-dominant and those starting from non-dominant to dominant separately using data pooled from all participants. The relationship between each phase duration wand the following three phase durations was tested using a mixed-effects linear regression separately for the three lags. Participant identity was included as a random variable in the model. $R^2$ was used to examine the explained variance of each model, whereas the Akaike (AIC) and Bayesian Information Criterion (BIC) were used for model comparison.

For the intra-modal consistency analysis, transition matrices, containing the conditional probabilities for transitions between perceptual alternatives, were constructed from the perceptual reports using the method described in Denham, et al. [8]. Transition matrices had three rows and columns, one for each perceptual alternative. Each element represents the conditional probability to switch from the starting percept (column) to the percept assigned to the row. A number of transition matrices were built. Firstly, all the data was pooled to calculate a global transition matrix. Next, transition matrices were calculated for each modality separately. Next, transition matrices were calculated separately for each modality for each participant, which will be referred as a participant transition matrix. Finally, transition matrices were also constructed for each block. Denham, et al. [8] showed that unobserved transitions from a transition matrix can be estimated from a transition matrix one above in the hierarchy. For example, an unobserved transition in a block transition matrix can be substituted with the corresponding value from the participant transition matrix.

**Multi-dimensional outliers in the correlations**

---

Multi-dimensional outliers that can influence the correlation were identified using the following tests and criteria: Mahalanobis distance (13.28, Barnett & Lewis, 1994), Cook distances (1, Cook & Weisberg, 1994), and Centered Leverage values (three times the fraction of the number of tested variables and sample size, which is .182 in our case, Stevens, 2012).

In the Neutral condition, no outliers were indicated by the Mahalanobis (max = 6.314) and Cook (max = 0.768) distances. However, there was a Centered Leverage value higher than

the threshold (0.197). Without this possible influential multi-dimensional outlier, the correlation would be non-significantly higher than the one reported in the main text ($r$(32) = .577, $p$ = .001, $z$ = 0.640, $p$ = .522). In the Hold condition similarly to the neutral, the Mahalanobis (max = 7.833) and Cook (max = 0.155) distances indicated no multi-dimensional outliers, but one participant had a Centered Level value higher than the threshold (0.245). The correlation without this participant would be non-significantly lower than the one reported in the main text ($r$(32) = .478, $p$ = .006, $z$ = -0.380, $p$ = .704). In the Switch condition, only the Cook distance indicated no outliers (max = .868), but one multi-dimensional outlier was indicated by both the participant's the Mahalanobis distance (15.659) and the Centered Leverage value (0.489). The correlation without this participant would be non-significantly lower than the one reported in the main text ($r$(32) = .598, $p$ < .001, $z$ = -0.170, $p$ = .865).

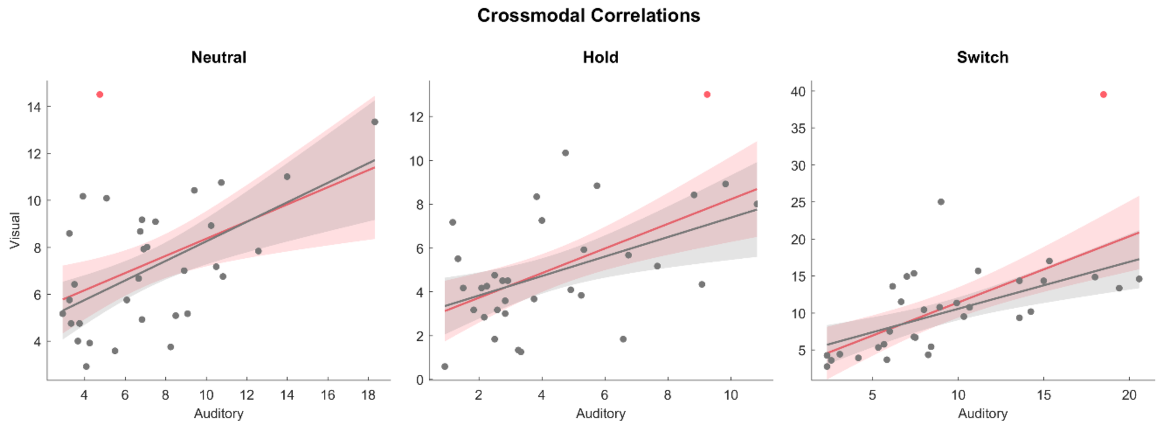


***Figure SM1.*** *Correlations in the number of perceptual switches across modalities, separately for each condition. The red dot indicates the outlier participant, while grey dots indicate other participants; grey (pink) shading indicates 95% confidence intervals of the slope of the regression line with (without) the outlier participant.*

Barnett, V., & Lewis, T. (1994). Outliers in statistical data (Vol. 3). New York: Wiley.

Cook, R. D., & Weisberg, S. (1982). Residuals and influence in regression.

Stevens, J. P. (2012). Applied multivariate statistics for the social sciences. Routledge.

**Further consideration of the successive phase correlations**

---

Although the correlations we find are larger than those reported previously [9,10], much of the difference can be ascribed to the use of the $\log_{10}$-correction. The plots in Figure SM2 were created by pooling all phase durations from all participants. This resembles the method used by Barniv and Nelken [9] and is different from the permutation-based method we adopted in the main text. The non-corrected scatterplots are highly similar to the ones reported by Barniv and Nelken [9]. It is also very clear that the non-corrected data does not meet the linearity and heteroscedasticity assumptions of Pearson correlation, whereas the $\log_{10}$-corrected data does. Once more the importance of the log-normal distribution is demonstrated, providing strong support for a distributed system of independent processes underlying perceptual switching.

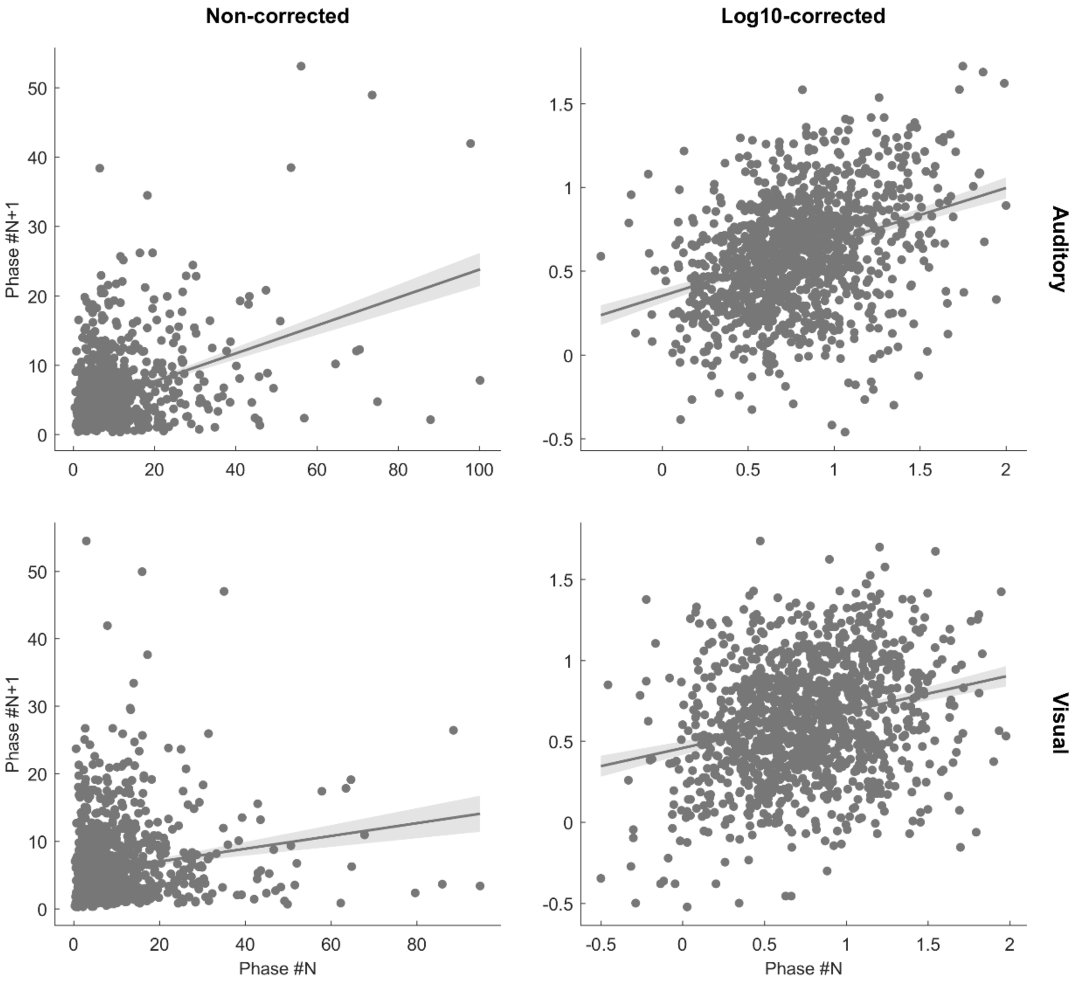


***Figure SM2.*** *Successive phase correlations of the dominant/non-dominant transitions at lag 1 separately for the auditory (top panels) and visual (bottom panels) modalities. On the left side, data was plotted without any correction, whereas on the right side, $log_{10}$-corrected data are depicted. 95% confidence intervals are indicated by shading.*

**Experiment 2**

---

In this section we describe a second experiment, just like the first, in which participants were asked to categorise their perceptions according to three instead of two perceptual alternatives. The method is mostly the same as that described for the first experiment, so here we only include details where the method differs.

***Experimental Design***

Table SM1 illustrates the time course of an experimental session.

| **Stage** | **Activity** | **Description** |
|---|---|---|
| 1 | Preliminary steps | Consent, handedness questionnaire, hearing test |
| 2 | Training<br>- Visual task<br>- Auditory task | Response categories<br>LEFT, RIGHT, COMBINED)<br>INTEGRATED, SEGREGATED, COMBINED |
| 3 | Test Condition 1: Neutral | 8 stimulus blocks: VVVVAAAA (AAAAVVVV) |
| 4 | Supplementary activity | Ego-resiliency questionnaire, (creativity questionnaire, Stroop task) |
| 5 | Test Condition 2: Hold (Switch) | 8 stimulus blocks: VVVVAAAA (AAAAVVVV) |
| 6 | Supplementary activity | Creativity questionnaire (Stroop task, ego-resiliency questionnaire) |
| 7 | Test Condition 3: Switch (Hold) | 8 stimulus blocks: VVVVAAAA (AAAAVVVV) |
| 8 | Supplementary activity | Stroop task (ego-resiliency questionnaire, creativity questionnaire) |

***Table SM1.*** *Experimental design, showing the eight stages in an experimental session. The order of the following were counterbalanced across participants: a) modality ordering of stimulus blocks, VVVVAAAA or AAAAVVVV, b) biased test conditions Hold/Switch in stages 5 and 7, c) supplementary task order ego-resiliency/creativity/Stroop in stages 4, 6 and 8.*

**Participants**

A total of 51 adults participated in this study. The study was run at two separate locations (Hungary: Research Centre for Natural Sciences of the Hungarian Academy of Sciences (RCNS), 31 adults, 21 females, $M_{age}$ = 21.48, $SD_{age}$ = 2.08; U.K.: University of Plymouth (UoP), 20 adults, 13 females, $M_{age}$ = 21.5, $SD_{age}$ = 3.05).

*Training*

***Visual Task***

Participants were instructed to report LEFT if they perceived the front face of the rotating structure-from-motion sphere moving leftwards, and RIGHT if they perceived the front face of the rotating sphere moving rightwards. It is also possible to perceive two half-spheres, moving in opposite directions[11,12]; participants were instructed to report COMBINED if they perceived this possibility.

These three interpretations were demonstrated to participants using disambiguated examples. LEFT (RIGHT) was disambiguated as explained before by reducing the luminance of the right-(left-) ward moving dots. The COMBINED interpretation was disambiguated with coloured dots; all of the dots moving leftwards were coloured green and those moving rightwards, yellow. The “Enter”, “Shift” and “Ctrl” keys located on the right-hand side of a standard computer keyboard were used as response keys, with key-response assignment counterbalanced across participants; see Figure SM3.

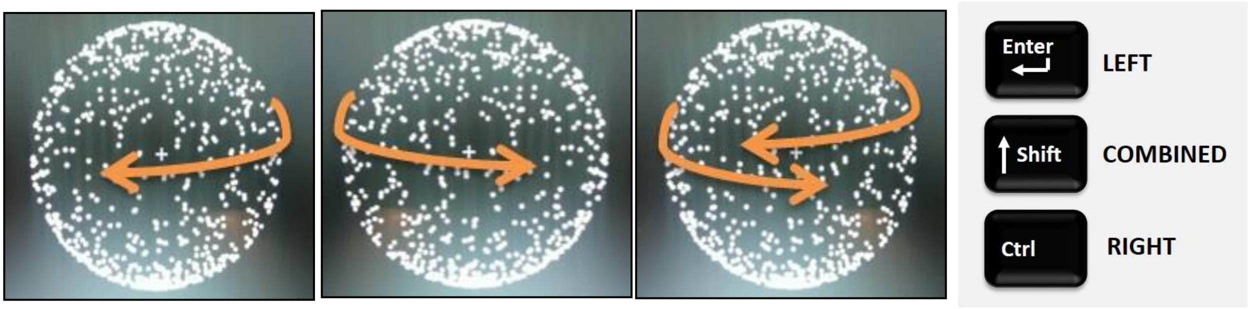


***Figure SM3.*** *Mnemonics and key assignments for categorising the ambiguous structure-from-motion stimulus.*

***Auditory Task***

The tone sequence consisting of a repeating LHL_ pattern can be perceived in different ways; see Figure SM4. Participants were instructed to respond INTEGRATED, if they perceived all tones as belonging together, and SEGREGATED if they perceived the tones separating into two streams of sounds, one containing only high the other only low tones. It is also possible to perceive the tone sequence with the H tones and some of the L tones in one stream and the other L tones in another stream; LH__ with __L_ or _HL_ with L___ [13,14]. Participants were also told about these possibilities and instructed to report COMBINED if they perceived the tone sequence in this way.

These three interpretations were demonstrated to participants using disambiguated examples. INTEGRATED (SEGREGATED) was disambiguated as explained before by reducing (increasing) the frequency difference between the L and H tones. To demonstrate the COMBINED percept, participants were presented with examples in which the intensity and timbre of the background stream (__L_ or L___ ) was modified (with frequency difference 4 semitones); intensity was reduced by 18dB, timbre was changed by adding harmonics 2-8 (with equal weight) to the fundamental. The corresponding mnemonics shown in Figure SM3 were used by way of explanation and later as reminders. The “Enter”, “Shift” and “Ctrl”

keys located on the right-hand side of a standard computer keyboard were used as response keys, with key-response assignment counterbalanced across participants.

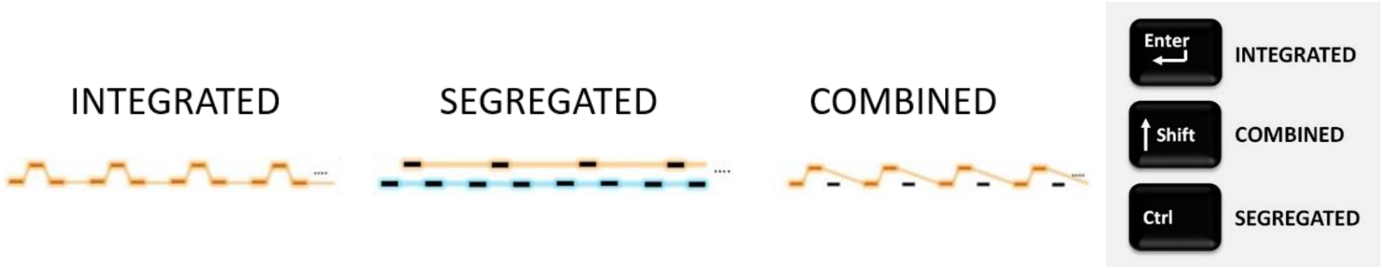


***Figure SM4.*** *Mnemonics and key assignments for categorising the ambiguous tone sequences.*

Training and testing procedures were the same as those described for the experiment in the main paper.

**Results for Experiment 2**

---

**Condition and modality**

The influence of the attentional manipulation and modality on the average number of switches was assessed using a 2 (modality: visual, auditory) x 3 (condition: Neutral, Hold, Switch) repeated-measures analysis of variance (rmANOVA), with Greenhouse-Geisser sphericity correction. The main effect of condition was significant ($F(2,56) = 35.841$, $p < .001$, $\eta^2_{partial} = .561$). This shows that participants were able to bias their perception according to task instructions. Neither the effect of modality ($F(1,28) = 3.425$, $p = .078$, $\eta^2_{partial} = .109$) nor the modality/condition interaction ($F(2,56) = 2.101$, $p = .137$, $\eta^2_{partial} = .070$) were significant (Figure SM5). Thus, participants switched similarly across modalities and the effect of

attentional bias was similar for the two modalities.

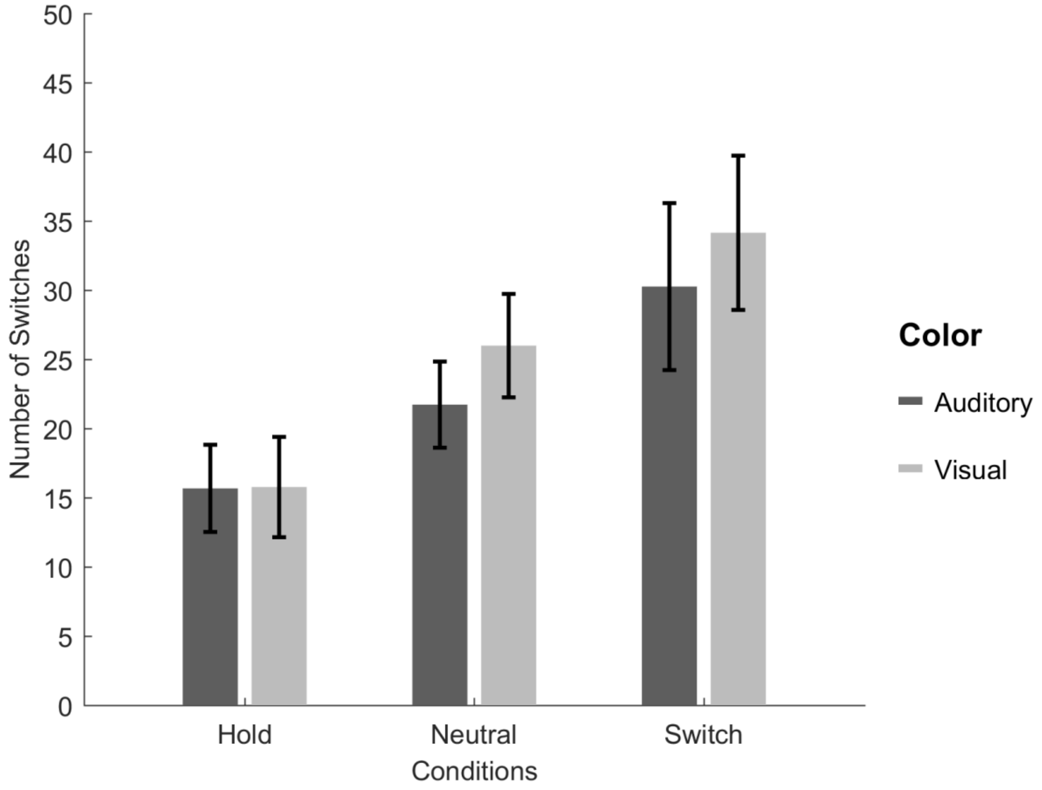


***Figure SM5.*** *Mean number of perceptual switches in each condition and modality. Error bars indicate 95% confidence intervals.*

While the categories used in the first experiment remained dominant, participants reported all three perceptual alternatives; see Figure SM6 for the mean proportions of each.

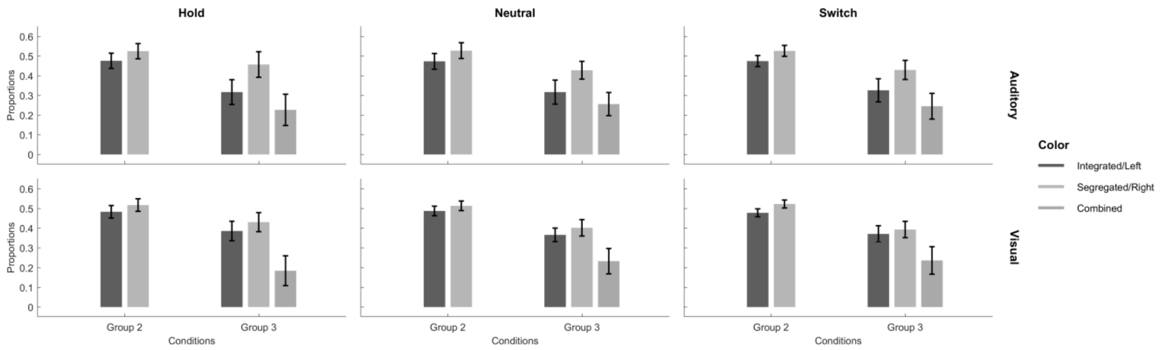


***Figure SM6.*** *Mean proportions for each perceptual category in each condition and modality. Error bars indicate 95% confidence intervals.*

## Correlations across modalities

Cross-modal relationships between the number of switches were tested with Pearson correlations separately for each condition (Figure SM7). Correlations between the two modalities were significant in the Neutral ($r(29) = .396$, $p = .034$, $CI_{95} = .034$-.666), Hold ($r(29) = .620$, $p < .001$, $CI_{95} = .327$-.804), and Switch conditions ($r(29) = .621$, $p < .001$, $CI_{95} = .330$-.805). The correlation coefficients observed for the Hold ($z = -1.104$, $p = .270$) and Switch ($z = -1.114$, $p = .265$) conditions did not differ from the Neutral condition. No significant correlations were found between the number of switches in the Neutral condition and measures in the creativity, ego-resiliency, and Stroop tasks.

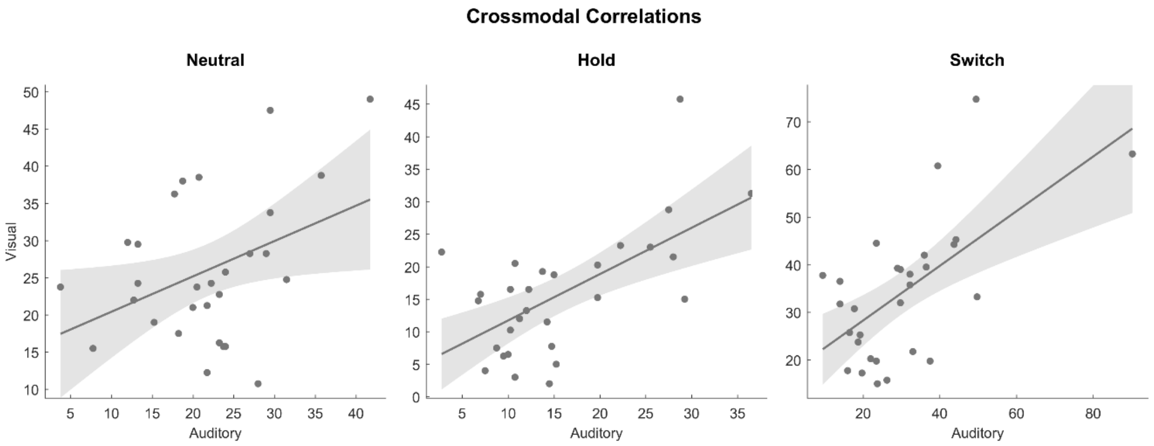


***Figure SM7.*** *Correlations in the number of perceptual switches across modalities, separately for each condition; 95% confidence intervals of the regression line indicated by shading.*

## Individual consistency

To explore individual consistency in switching across modalities, percepts were first reorganized into dominant/middle dominant/non-dominant categories (i.e. percepts were relabeled block-by-block according to their dominance to allow comparisons between the two modalities). Participant transition matrices[8] were then constructed from the auditory and visual Neutral condition data. Intra-modal consistency was measured as the Kullback-Leibler (K-L)[15] divergence between participants' auditory and visual transition matrices. Inter-participant consistency was measured by comparing the K-L distances between a participant's transition matrices and the transition matrices of all other participants. The distributions of intra-modal and inter-participant distance measures were compared using a left-tailed Wilcoxon's Rank Sum test. (One-sided significance testing was chosen, because the hypothesis was that participant intra-modal distances should be smaller than inter-participant distances). The result of the test ($z$ = -0.509, $p$ = .306) indicates that participants' perceptual switching behaviour was not more similar across the two modalities ($M$ = .146,

$CI_{95}$ = .077-.216) than the variation across participants (*M* = .181, $CI_{95}$ = .168-.192). In short, in contrast to the first experiment, participants did not respond consistently within and across modalities.

**Comparison of the distributions of phase durations across modalities**

Raw phase durations from the Neutral condition only were pooled across participants separately for each modality. First, we tested whether the distribution of the phase durations was gamma or lognormal. Examination of Q-Q-plots [6] indicated that the lognormal distribution fits the data better than the gamma distribution (Figure SM8).

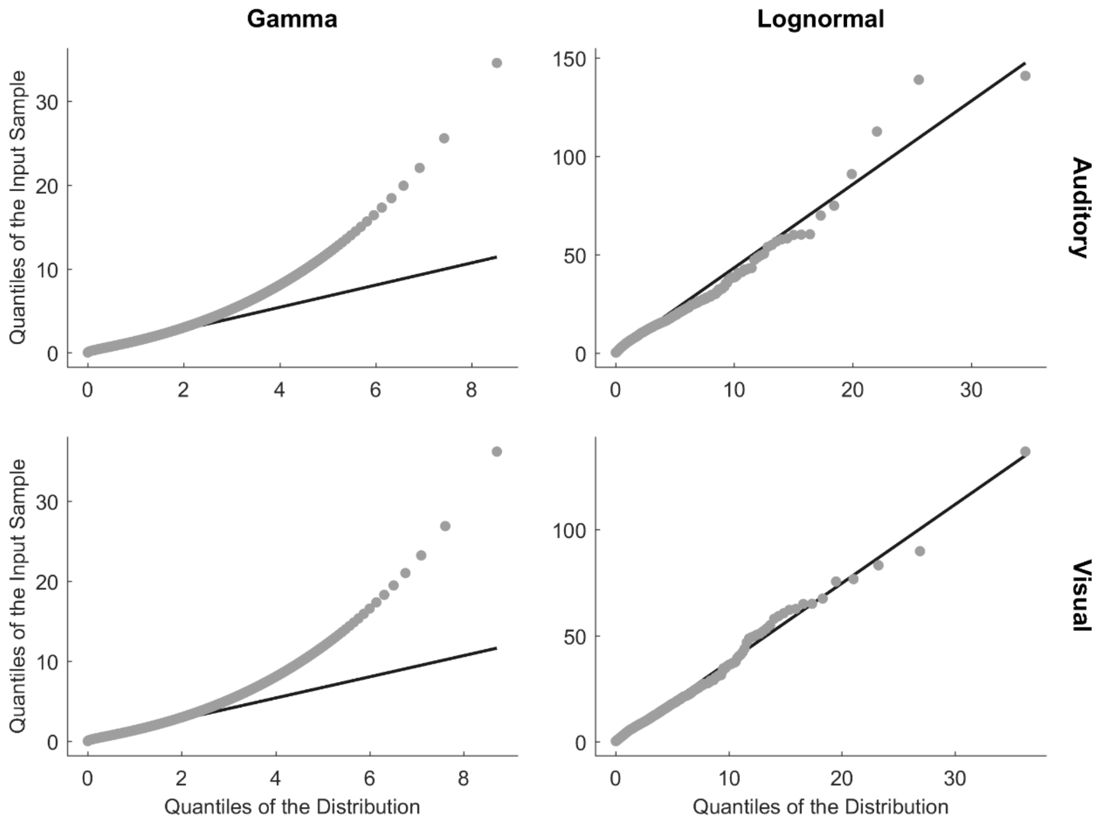

***Figure SM8.*** *QQ-plots of gamma (left) and lognormal (right) distributions for phase durations from the Neutral conditions in the auditory (upper row) and visual (lower row) modalities.*

Phase distributions from the two modalities were first compared to each other using a Two-Sample Kolmogorov-Smirnov test. The result indicates that auditory and visual distributions were significantly different from each other ($D = 0.102$, $p = .003$). Second, the mu and sigma parameters of the lognormal distributions were calculated for phase durations separately for the auditory and visual modalities with 95% confidence intervals (Figure SM9). Conversely to what was observed in the first experiment, the confidence intervals of the sigma parameter overlap across the two modalities and the confidence intervals of the mu parameter do not. This shows that although phase durations in the auditory and visual modalities can both be described by the same type of distribution (log-normal), the details of the distributions are different.

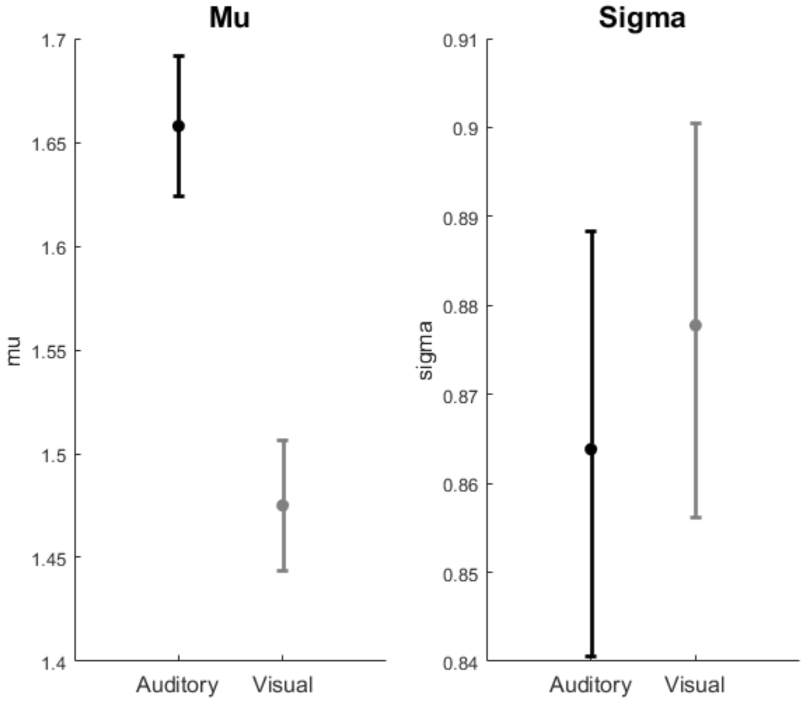

***Figure SM9.*** *Mu (left) and Sigma (right) parameters of the lognormal distribution with 95% confidence intervals. In both subplots, the auditory modality is on the left side in black, and visual is on the right in grey.*

**Correlations between phases**

The relationships between successive phase durations (i.e. between the sequences of perceptual dominance durations) were tested with a mixed-level linear regression where participant identity was included as a random effect. Raw phase durations from the Neutral condition were $\log_{10}$-corrected in accordance with their log-normal distribution. This allowed us to meet not only the normality but also the linearity and heteroscedasticity assumptions of the test Similarly to the individual consistency analysis, percepts were relabeled based on their overall duration in a block-by-block basis as Dominant (having the highest phase duration in a block), Non-dominant (having the lowest phase duration in a block), and Middle Dominant (the one between the Dominant and Non-dominant categories). Correlations were tested in both modalities for only one lag for all possible combinations of the Dominant/Middle Dominant/Non-dominant categories. The same transitions (D/MD, MD/D, ND/D) were significant in both modalities (Table SM2). Further, the $R^2$, AIC, and BIC measures indicate a better model fit in the auditory than the visual modality.

| **Modality** | **Transition** | ***N*** | ***R*²** | **AIC** | **BIC** | **Unstandardized *b*** | ***t*** | ***r*** |
|---|---|---|---|---|---|---|---|---|
| Auditory | D/MD | 668 | 13.92% | 406.88 | 424.90 | .174 (.102-.246) | 4.759*** | .373 |
| | D/ND | 271 | 0.81% | 227.15 | 241.56 | .085 (-.027-.198) | 1.489 | .090 |
| | MD/D | 680 | 12.93% | 415.34 | 433.43 | .185 (.112-.258) | 4.969*** | .360 |
| | MD/ND | 188 | 2.73% | 152.04 | 164.99 | .156 (.022-.291) | 2.295* | .165 |
| | ND/D | 257 | 2.79% | 230.07 | 244.27 | .167 (.046-.288) | 2.717** | .167 |
| | ND/MD | 198 | 0.56% | 189.00 | 202.15 | .082 (-.071-.235) | 1.057 | .075 |
| Visual | D/MD | 882 | 12.89% | 640.07 | 659.20 | .153 (.090-.216) | 4.774*** | .359 |

| | | | | | | | | |
|---|---|---|---|---|---|---|---|---|
| | D/ND | 322 | 17.56% | 212.13 | 227.23 | .054 (-.042-.150) | 1.111 | .419 |
| | MD/D | 892 | 13.95% | 674.93 | 694.11 | .181 (.114-.248) | 5.302*** | .374 |
| | MD/ND | 230 | 28.47% | 157.55 | 171.30 | .028 (-.086-.142) | 0.491 | .534 |
| | ND/D | 327 | 1.24% | 296.85 | 312.01 | .118 (.003-.232) | 2.022* | .111 |
| | ND/MD | 223 | 12.93% | 170.20 | 183.83 | -.019 (-.153-.114) | -0.286 | .360 |

***Table SM2.*** *Relationship between successive phases in the auditory and visual modalities. "Transition" refers the Dominant/Non-Dominant (D/ND), Dominant-Middle Dominant (D/MD), Middle Dominant/Dominant (MD/D), Middle Dominant/Non-dominant(MD/ND), the Non-Dominant/Dominant (ND/D), and Non-Dominant/Middle Dominant (ND/MD) transitions. $R^2$ refers to the explained variance of the model, whereas AIC and BIC refer to the Akaike or Bayesian Information Criterion, respectively. "Unstandardized b" refers to the slope of the model with $CI_{95}$ values included in parenthesis. "t" refers to the t-test examining the slope's difference from zero and asterisks are indicating the level of significance (*** p < .001, ** p < .01, * p < .05). "r" refers to the correlation coefficient between the two phases estimated from the $R^2$.*

**Conclusion**

The results from the second experiment were broadly consistent with those of first experiment. However, participants found the auditory three-alternative task far more difficult than the visual one, as evidenced by the number of participants incorrectly categorising the disambiguated segments appended to the end of the test blocks. This difference may have caused some of the differences observed in the cross-modal comparisons.

## References


1 Lansbergen, M. M., Kenemans, J. L. & van Engeland, H. Stroop interference and attention-deficit/hyperactivity disorder: A review and meta-analysis. *Neuropsychology* **21**, 251-262, doi:10.1037/0894-4105.21.2.251 (2007).

2 Block, J. & Kremen, A. M. IQ and ego-resiliency: Conceptual and empirical connections and separateness. *J Pers Soc Psychol* **70**, 349-361, doi:10.1037/0022-3514.70.2.349 (1996).

3 Farkas, D. & Orosz, G. Ego-Resiliency Reloaded: A Three-Component Model of General Resiliency. *Plos One* **10**, doi:10.1371/journal.pone.0120883 (2015).

4 Batey, M. & Furnham, A. Creativity, intelligence, and personality: A critical review of the scattered literature. *Genetic, social, and general psychology monographs* **132**, 355-429, doi:10.3200/MONO.132.4.355-430 (2006).

5 Fisher, R. A. Frequency distribution of the values of the correlation coefficient in samples of an indefinitely large population. *Biometrika* **10**, 507-521 (1915).

6 Wilk, M. B. & Gnanadesikan, R. Probability plotting methods for the analysis for the analysis of data. *Biometrika* **55**, 1-17 (1968).

7 Massey, F. J. The Kolmogorov-Smirnov Test for Goodness of Fit. *Journal of the American Statistical Association* **46**, 68-78 (1951).

8 Denham, S. L. *et al.* Characterising switching behaviour in perceptual multi-stability. *J Neurosci Meth* **210**, 79-92, doi:10.1016/j.jneumeth.2012.04.004 (2012).

9 Barniv, D. & Nelken, I. Auditory Streaming as an Online Classification Process with Evidence Accumulation. *Plos One* **10**, doi:10.1371/journal.pone.0144788 (2015).

10 van Ee, R. Stochastic variations in sensory awareness are driven by noisy neuronal adaptation: evidence from serial correlations in perceptual bistability. *J Opt Soc Am A* **26**, 2612-2622 (2009).

11 Hol, K., Koene, A. & van Ee, R. Attention-biased multi-stable surface perception in three-dimensional structure-from-motion. *J Vision* **3**, 486-498, doi:10.1167/3.7.3 (2003).

12 Chen, X. C. & He, S. Local factors determine the stabilization of monocular ambiguous and binocular rivalry stimuli. *Current Biology* **14**, 1013-1017, doi:10.1016/j.cub.2004.05.042 (2004).

13 Denham, S. L., Gymesi, K., Stefanics, G. & Winkler, I. Multistability in auditory stream segregation: the role of stimulus features in perceptual organisation. *Journal Learning & Perception* **2**, 73-100, doi:10.1098/rstb.2011.0359 (2013).

14 Denham, S. L. *et al.* Stable individual characteristics in the perception of multiple embedded patterns in multistable auditory stimuli. *Front Neurosci-Switz* **8**, doi:10.3389/fnins.2014.00025 (2014).

15 Kullback, S. & Leibler, R. A. On information and sufficiency. *Annals of Mathematical Statistics* **22**, 79-86 (1951).
